# Safety and efficacy of low-dose and long-course tirofiban in large hemispheric infarction

**DOI:** 10.3389/fneur.2022.987859

**Published:** 2022-09-09

**Authors:** Yinqin Hu, Qian Xiao, Zhizhen Shi, Yangbo Hou, Zhen Chen, Jiwei Cheng, Guoyi Li

**Affiliations:** Department of Neurology, Putuo Hospital, Shanghai University of Traditional Chinese Medicine, Shanghai, China

**Keywords:** large hemispheric infarction, tirofiban, clopidogrel, antiplatelet aggregation, acute ischemic stroke

## Abstract

**Background:**

The clinical efficacy and safety of tirofiban in the treatment of large hemispheric infarction (LHI) remain controversial.

**Methods:**

This study prospectively enrolled patients with acute LHI who were admitted to Putuo Hospital affiliated with Shanghai University of Traditional Chinese Medicine from June 2021 to December 2021. The patients were randomly assigned to the tirofiban group [3–4 μg/(kg·h)] or control group (clopidogrel 75 mg/d).

**Results:**

A total of 71 patients with acute LHI were selected: 36 in the tirofiban group and 35 in the control group. The reduction of the NIHSS score in the tirofiban group was 2.92 ± 9.31 at discharge, and that of the control group was −3.23 ± 12.06 (*p* = 0.021, *OR*, 0.006; 95% *CI*, 0.004–0.008). Six patients (16.7%) in tirofiban group and 14 patients (40%) in control group died during hospitalization (*p* = 0.029, *OR*, 0.300; 95% *CI*, 0.099–0.908). There was significant difference in Modified Rankin Scale (mRS) 5–6 scores at 90 days between the two groups (*p* = 0.023, *OR*, 0.327; 95% *CI*, 0.124–0.867). However, there was no significant difference in mRS 0–1 (*p* = 0.321, *OR*, 0.972; 95% *CI*, 0.920–1.027), mRS 2 (*p* = 0.572, *OR*, 2.00; 95% *CI*, 0.173–23.109), mRS 3 (*p* = 0.225, *OR*, 2.214; 95% *CI*, 0.601–8.161), or mRS 4(*p* = 0.284, *OR*, 1.859; 95% *CI*, 0.593–5.825) scores between the two groups. There was no difference in symptomatic intracranial hemorrhage (*p* = 0.29, *OR*, 0.305; 95% *CI*, 0.030–3.081), asymptomatic intracranial hemorrhage (*p* = 0.123, *OR*, 0.284; 95% *CI*, 0.053–1.518). There was a significant difference in systemic bleeding events during hospitalization (*p* = 0.044, *OR*, 0.309; 95% *CI*, 0.096–1.000).

**Conclusions:**

Low-dose and long-course tirofiban treatment may significantly improve the early neurological function and reduce the in-hospital mortality in LHI patients. Meanwhile, tirofiban does not increase the risk of any type of bleeding events.

## Introduction

Stroke is one of the main causes of death and disability worldwide. The lifelong risk and disease burden of stroke are as high as 39.3% in China, which ranks first globally (1). Ischemic stroke is the main type of stroke, accounting for 81.9% of all strokes (2). Large hemispheric infarction (LHI) refers to an infarction of more than 2/3 of the blood supply area of the middle cerebral artery, with or without the infarction of the anterior cerebral artery/posterior cerebral artery, and the incidence rate is ~10–20/100,000 per year (3). LHI is characterized by severe symptoms, rapid progression, a high risk of bleeding, and high mortality.

Antiplatelet drugs play an extremely key role in the prevention and treatment of acute ischemic stroke. Tirofiban is a non-peptide intravenous antiplatelet drug that can reversibly antagonize the platelet GP II B/III A receptor and inhibit the binding of fibrinogen to the platelet GP II B/III A receptor, and can improve the cerebral circulation. Tirofiban is mainly used in the antithrombotic treatment of patients with acute non-ST-segment elevation coronary syndrome. In recent years, tirofiban has been recommended by some studies and guidelines as an important short-term antithrombotic therapy for progressive stroke with arteriolar occlusion and perioperative patients undergoing endovascular intervention (4–7). However, the clinical efficacy and safety of tirofiban in the treatment of LHI have not been reported. The purpose of this study was to explore the efficacy and safety of low-dose and long-course tirofiban in the treatment of acute LHI to provide a clinical reference.

## Methods

### Trial design and patients

**Figure 1:**
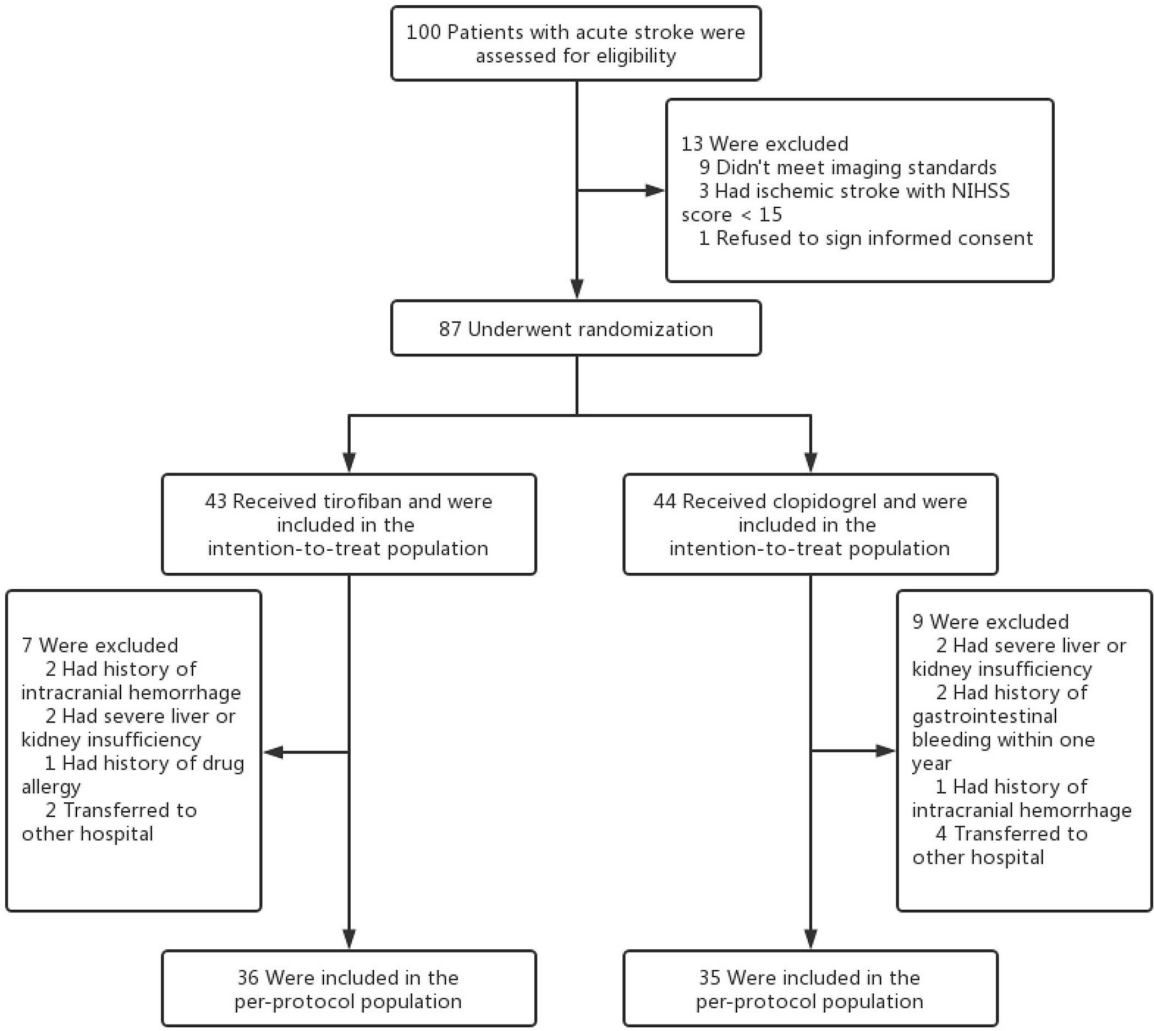
Flow chart of the study.

This study is a prospective cohort study. Patients with acute LHI who were admitted to Putuo Hospital affiliated with the Shanghai University of Traditional Chinese Medicine from June 2021 to December 2021 were randomly divided into two groups: tirofiban group or control group (Figure 1). The study was approved by the Ethics Committee of Putuo Hospital Affiliated with Shanghai University of Traditional Chinese Medicine and was in accordance with the Declaration of Helsinki. Informed consent was obtained from all participants or their legal guardians.

The inclusion criteria were as follows: (1) patients who met the diagnostic criteria of LHI in the 2017 Chinese Expert Consensus on Monitoring and Treatment of Large Cerebral Infarction in the cerebral Hemisphere (8); (2) patients older than 18; and (3) patients or their legal guardians signed informed consent. The exclusion criteria were as follows: patients who (1) had intracranial hemorrhage, intracranial tumors, intracranial aneurysms, arteriovenous malformations, and infections; (2) had coagulation disorders or bleeding tendency; (3) had a history of myocardial infarction and craniocerebral trauma within the last 3 months, gastrointestinal and urinary tract bleeding within 3 weeks, major surgery within 2 weeks, and a fracture within 1 week; (4) had a blood pressure >190/110 mmHg (1 mmHg = 0.133 kPa), a platelet count (PLT) < 100 × 109/L, a fasting blood glucose <2.7 mmol/L; (5) had severe cardiac, hepatic, and renal dysfunction; (6) were pregnant or lactating; (7) had a history of drug allergy; and (8) did not sign the informed consent.

After enrollment, the following data were collected: age, sex, stroke subtypes (such as the TOAST and OCSP classification), medical history (hypertension, diabetes, coronary heart disease, atrial fibrillation, hyperlipidemia, tobacco, and alcohol history), and the admission baseline data (systolic blood pressure, diastolic blood pressure, NIHSS score, and Modified Rankin Scale (mRS) score).

The CT scans were performed 24 h and 7 days after treatment to assess the hemorrhage conversion. In addition, neurological examinations and routine blood, fecal, and urine test were performed every day during the patient's hospitalization to determine the safety of the therapy. The investigators followed up with the subjects by telephone 90 days after discharge from the hospital to assess their neurological function and living ability using mRS scores.

### Treatment

Patients in the tirofiban group received 3–4 μg/(kg·h) tirofiban through intravenous injection; those in the control group were given 75 mg/d clopidogrel through nasal feeding. The patients in both groups continued receiving assigned medications until discharge. In addition, the patients in both groups received the same basic treatment [atorvastatin for plaque stabilization and lipid regulation, mannitol dehydration for intracranial pressure reduction, and edaravone for brain tissue protection]. After discharge, the patients continued to take oral antiplatelet medication, rehabilitation, and nursing treatment according to local standards. The follow-up and data collection were continued for 3 months.

The main experimental drugs used in this study were as follows: (1) Tirofiban (trade name: *Xinweining*; Wuhan Grand Pharmaceutical Group Co., LTD. (China); National Drug approval H20041165); (2) Clopidogrel (trade name: Taijia; Shenzhen Salubris Pharmaceuticals Co., Ltd.; National drug approval word H20000542).

### Outcomes

The primary efficacy outcome measure was if there was an early improvement in the patient's neurological function, which was mainly based on the improvement in the patient's NIHSS score at discharge. The secondary efficacy outcome measures included mortality during hospitalization and the long-term neurological outcome which was evaluated by the mRS scores at 90 days. According to the severity of stroke, the patients were divided into (1) mild stroke (mRS scored 0–1); (2) moderate stroke (mRS scored 2–3); (3) severe stroke (mRS scored 4–5); and (4) fatal stroke (mRS scored 6).

**Figure 2:**
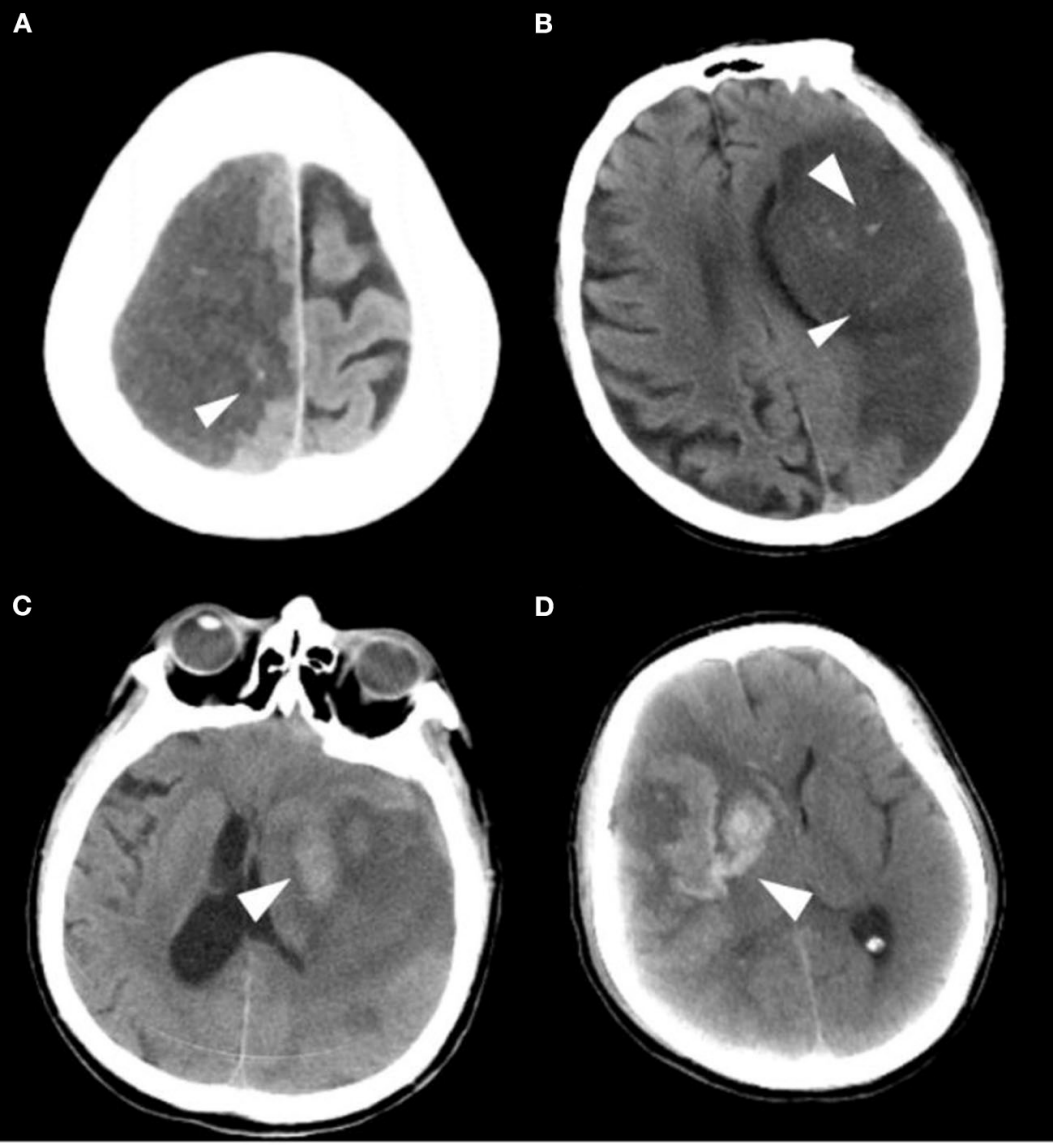
Imaging classification of the hemorrhagic transformation. **(A)** HI 1: Small petechiae along the margins of the infarct. **(B)** HI 2: More confluent petechiae within the infarcted area, but without space-occupying effect. **(C)** PH 1: Blood clot not exceeding 30% of the infarcted area with some mild space-occupying effect. **(D)** PH 2: Dense blood clot(s) exceeding 30% of the infarct volume with significant space-occupying effect.

The primary safety outcome was the development of any intracranial hemorrhage events during hospitalization [as defined by the ECASS (9) criteria: HI1, hemorrhagic infarction type 1; HI2, hemorrhagic infarction type 2; PH1, parenchymal hemorrhage type 1; PH2, parenchymal hemorrhage type 2], as shown in Figure 2. Symptomatic intracranial hemorrhage was defined as blood at any site in the brain on the CT scan (as assessed by the CT reading panel, independently of the assessment by the investigator), documentation by the investigator of clinical deterioration, or adverse events indicating clinical worsening (e.g., drowsiness and increase of hemiparesis) or causing a decrease in the NIHSS score of 4 or more points. Asymptomatic intracranial hemorrhage (ASICH) was defined as little hemorrhage at any site of the brain on the CT scan (as assessed by the CT reading panel, independently of the assessment by the investigator), which did not conform to any criteria of the above SICH definition.

The secondary safety outcomes also included any non-intracranial bleeding, deaths, and other serious adverse events during the trial.

### Statistical analysis

In this study, the IBM SPSS Statistics 25.0 software was used for statistical analysis, and Microsoft Office Excel was used to collect data. The efficacy and safety of the medications used in the two groups were analyzed. Categorical variables were expressed as numbers (percentages), while continuous variables were expressed as mean ± standard deviation (*SD*). When a baseline factor was a continuous variable with a normal distribution, the independent sample *T*-test was used. If the data did not conform to a normal distribution, a non-parametric test (Mann–Whitney *U* rank-sum test) was used. When any of the baseline factors were classified variables, the chi-square (χ^2^) test and Fisher's exact test were used. During hospitalization, the all-cause deaths that occurred in the patients were counted and estimated by Kaplan–Meier plots. All tests were two-sided, and the significance level of the *p*-values was set at 0.05.

## Results

### Patient characteristics

Overall, from June 2021 to December 2021, we enrolled a total of 36 patients who received low-dose and long-course [median (quartile range), 8.5 (5–10); mean ± *SD*, 8.33 ± 4.58 days) treatment with tirofiban and 35 patients who received clopidogrel. After enrollment, the baseline information and experimental data of the patients were recorded, and all of the patients were followed up for 90 days. The ages of tirofiban group and control group were 72.57 ± 12.39 and 73.89 ± 9.82, respectively (*p* = 0.665). There were 29 men (80.6%) in tirofiban group and 18 men (51.4%) in control group (*p* = 0.009). The baseline data of the diseases and habits and the clinical data at admission were comparable between the two groups, except for the history of coronary heart disease (*p* = 0.013) and the history of smoking (*p* = 0.003). The etiological classifications of TOAST in the two groups were comparable too, there were 30 (83.3%) patients with LAA and 6 (16.7%) patients with CE in the tirofiban group compared with 29 (82.9%) patients with LAA and 6 (17.1%) patients with CE patients in the control group (LAA, *p* = 0.957; CE, *p* = 1.000). At the same time, all the patients in the two groups belonged to the complete anterior circulation occlusion according to the OCSP classification (Table 1).

**Table 1:** Characteristics of the patients at baseline.

**Characteristics**	**Tirofiban (*n* = 36)**	**Control** **(*n* = 35)**	***P***
**Sociodemographic characteristics**			
Male, *n* (%)	29 (80.6)	18 (51.4)	0.009
Age (y, mean ± SD)	72.57 ± 12.39	73.89 ± 9.82	0.665
**Diseases and bad habits history**, ***n*** **(%)**			
Hypertension	29 (80.6)	28 (80)	0.953
Diabetes mellitus	14 (38.9)	16 (45.7)	0.561
Hyperlipidemia	12 (33.3)	8 (22.9)	0.327
Atrial fibrillation	9 (25)	12 (34.3)	0.391
Coronary heart disease	13 (36.1)	23 (65.7)	0.013
Smoking	17 (47.2)	5 (14.3)	0.003
Drinking	7 (19.4)	9 (25.7)	0.728
**Clinical data at admission (mean ±SD)**			
SBP (mmHg)	155.43 ± 16.78	155.60 ± 24.23	0.975
DBP (mmHg,)	85.86 ± 11.12	82.29 ± 12.53	0.239
NIHSS	22.97 ± 9.48	24.26 ± 8.43	0.244
FBG (mmol/L)	9.27 ± 4.18	10.57 ± 4.14	0.076
Uric acid (μmol/L)	301.42 ± 109.08	360.69 ± 144.42	0.092
TG (mmol/L)	1.17 ± 0.50	1.35 ± 1.18	0.273
Cholesterol (mmol/L)	4.33 ± 0.99	4.42 ± 1.21	0.106
LDL (mmol/L)	2.94 ± 0.80	2.85 ± 0.89	0.144
HDL (mmol/L)	1.12 ± 0.25	1.21 ± 0.36	0.198
HCY (mmol/L)	15.86 ± 9.99	17.07 ± 9.21	0.248
**Etiology classification, TOAST**, ***n*** **(%)**			
LAA	30 (83.3)	29 (82.9)	0.957
SAO	0 (0)	0 (0)	
CE	6 (16.7)	6 (17.1)	1.000
ODC	0 (0)	0 (0)	
UND	0 (0)	0 (0)	

SBP, systolic blood pressure; DBP, diastolic blood pressure; NIHSS, national institute of health stroke scale; FBG, fasting blood glucose; TG, triglyceride; TOAST, trial of org 10172 in acute stroke treatment; LDL, low-density lipoprotein; HDL, high density lipoprotein; HCY, homocysteine; LAA, large-artery atherosclerosis; SAO, small-artery occlusion; CE, cardioembolism; ODC, stroke of other determined cause; UND, stroke of undetermined cause.

## Outcomes

### Efficacy outcomes

#### National institute of health stroke scale, NIHSS

A significant improvement in NIHSS score at discharge was seen in tirofiban group compared with the baseline NIHSS score (20.00 ± 12.16 *vs*. 27.46 ± 12.74, *p* = 0.022, *OR*, 0.022; 95% *CI*, 0.019–0.025). And, there was a significant difference in NIHSS score reduction values at discharge between the tirofiban group and the control group (2.92 ± 9.31 *vs*. −3.23 ± 12.06, *p* = 0.021, *OR*, 0.006; 95% *CI*, 0.004–0.008) (Table 2).

**Table 2:** Efficacy and safety outcomes.

**Characteristics**	**Tirofiban (*n* = 36)**	**Control (*n* =3 5)**	***P***	***OR*** **(95% *CI*)**
**NIHSS (mean ±SD)**				
NIHSS at discharge	20.00 ± 12.16	27.46 ± 12.74	0.022	
NIHSS reduction value	2.92 ± 9.31	−3.23 ± 12.06	0.006	
**mRS score distribution at 90 d**, ***n*** **(%)**				
mRS 1	1 (2.8)	0 (0)	0.321	0.972 (0.920–1.027)
mRS 2	2 (5.6)	1 (2.9)	0.572	2.000 (0.173–23.109)
mRS 3	8 (22.2)	4 (11.4)	0.225	2.214 (0.601–8.161)
mRS 4	10 (27.8)	6 (17.1)	0.284	1.859 (0.593–5.825)
mRS 5–6	15 (41.7)	24 (68.6)	0.023	0.327 (0.124–0.867)
**Hemorrhagic risk**, ***n*** **(%)**				
SICH	1 (2.8)	3 (8.6)	0.29	0.305 (0.030–3.081)
ASICH	2 (5.6)	6 (17.1)	0.123	0.284 (0.053–1.518)
Systemic bleeding	5 (15.6)	12 (34.8)	0.044	0.309 (0.096–1.000)
**Mortality in hospital**, ***n*** **(%)**				
Total	6 (16.7)	14 (40)	0.029	0.300 (0.099–0.908)
<70	1 (7.1)	2 (28.6)	0.508	
70–80	5 (41.7)	7 (38.9)	0.879	
>80	0 (0)	5 (50)	0.016	

NIHSS, national institute of health stroke scale; mRS, modified rankin scale score; SICH, symptomatic intracranial hemorrhage; ASICH, asymptomatic intracranial hemorrhage.

#### In-hospital mortality

The in-hospital mortality was significantly lower in tirofiban group than in control group (16.7 *vs*. 40%, *p* = 0.029, *OR*, 0.300; 95% *CI*, 0.099–0.908). The subgroup analysis according to patient age showed that, in the tirofiban group, there were 6 patients died in those <80 years, and no patient died in those >80 years. In the control group, there were 9 patients died in those <80 years, and 5 patients died in those >80 years. The difference in mortality between the two groups came mainly from those patients >80 years (0 *vs*. 5, *p* = 0.016) (Table 2).

#### Modified rankin scale, mRS

**Figure 3:**
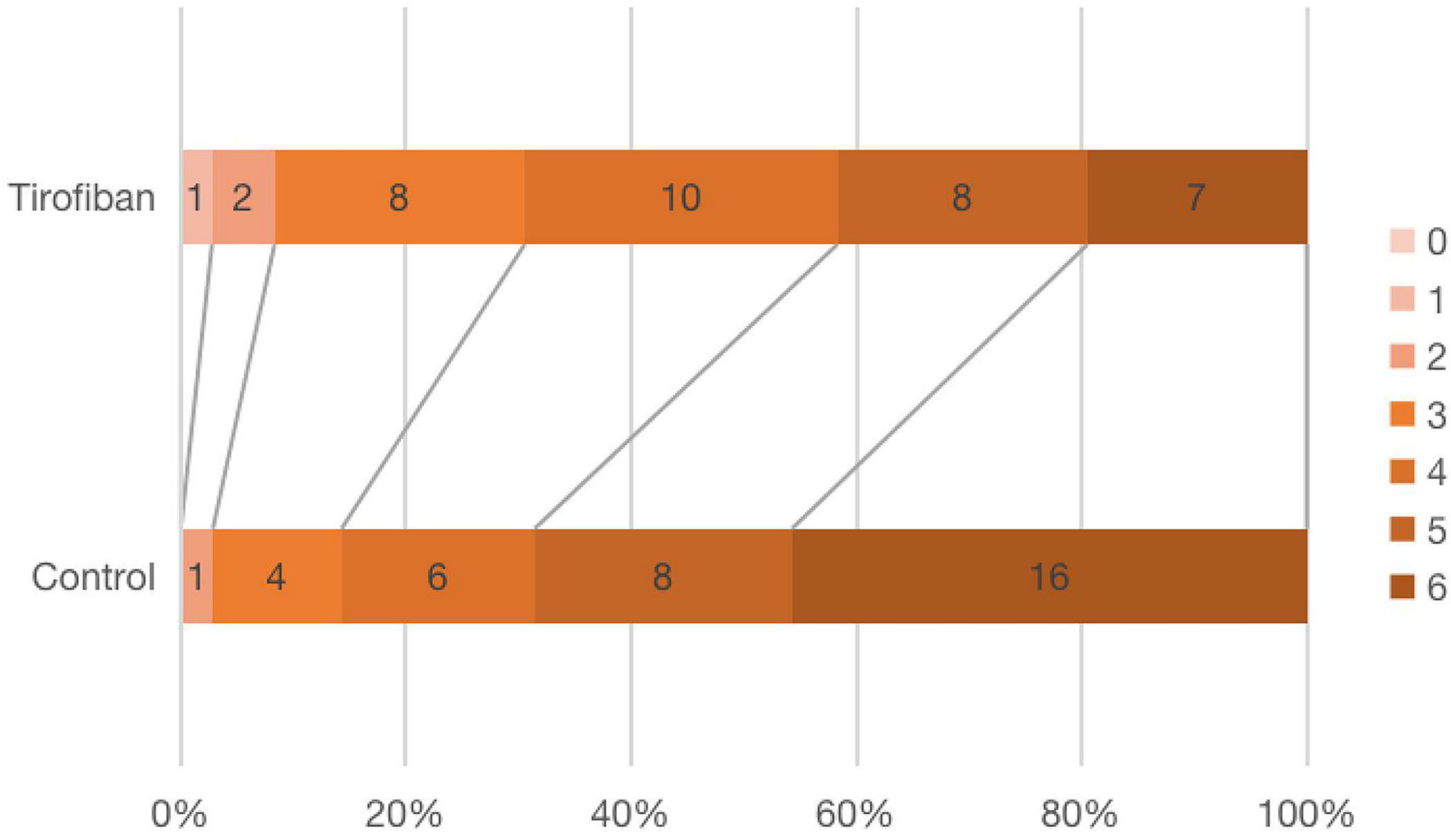
Distribution of the modified rankin scale (mRS) scores at 90 days.

Figure 3 showed the mRS scores distribution at 90 days in all patients. There was no difference in mRS 0–1 (*p* = 0.321, *OR*, 0.972; 95% *CI*, 0.920–1.027), mRS 2 (*p* = 0.572, *OR*, 2.00; 95% *CI*, 0.173–23.109), mRS 3 (*p* = 0.225, *OR*, 2.214; 95% *CI*, 0.601–8.161) or mRS 4 (*p* = 0.284, *OR*, 1.859; 95% *CI*, 0.593–5.825) scores between the two groups. There were significantly fewer severe disabilities (mRS = 5) and fatal strokes (mRS = 6) in tirofiban group than in control group (15 *vs*. 24, *p* = 0.023, *OR*, 0.327; 95% *CI*, 0.124–0.867) (Table 2).

### Safety outcomes

#### Intracranial hemorrhage

There were 3 patients with intracranial hemorrhage in tirofiban group 1 case of HI2, 1 case of PH1, and 1 case of PH2. In the control group, there were 9 patients with intracranial hemorrhage, 1 case of HI1, 5 cases of HI2, 2 cases of PH1, and 1 case of PH2. SICH occurred in 1 patient (2.8%) in tirofiban group and 3 patients (8.6%) in the control group; ASICH occurred in 2 patients (5.6%) in the tirofiban group, and 6 patients (17.1%) in the control group. Tirofiban did not increase the risk of symptomatic or asymptomatic intracranial hemorrhage compared with control group (SICH: *p* = 0.29, *OR*, 0.305; 95% *CI*, 0.030–3.081; ASICH: *p* = 0.123, *OR*, 0.284; 95% *CI*, 0.053–1.518) (Table 2).

### Other bleeding events

We recorded all kinds of non-intracranial bleeding events during hospitalization, such as gastrointestinal bleeding, urinary bleeding, and skin and mucosa bleeding. There were 5 cases of bleeding (15.6%) in the tirofiban group and 12 cases of bleeding (34.8%) in the control group. The analysis showed that tirofiban did not increase the risk of non-intracranial bleeding in patients with acute LHI (*p* = 0.044, *OR*, 0.309; 95% *CI*, 0.096–1.000) (Table 2).

## Discussion

Acute LHI is a devastating disease that is originally known as malignant middle cerebral artery infarction (MMI) (10, 11). MMI is characterized by severe symptoms, rapid progression, high risk of hemorrhage transformation, and high mortality. If not treated in time, severe complications such as cerebral edema, hemorrhage transformation, and cerebral herniation will occur in the patients and may endanger their lives. The previous studies have shown that the mortality rate of MMI is as high as 60.9–78%, and it is most commonly caused by cerebral herniations (10, 12). Even if patients survive after treatment, the severe neurological dysfunction will remain. The previous studies have shown that ~43–89% of MMI patients have mRS scores of 4–5 (12–14). Due to the poor antithrombotic effect of LHI and the occurrence of hemorrhagic transformation, it is necessary to seek a safe and effective antithrombotic program.

Tirofiban is a non-peptide intravenous antiplatelet drug that can reversibly inhibit the binding of fibrinogen to the platelet GP II B/III A receptor and improve the cerebral circulation. The drug is mainly used in patients with acute non-ST-segment elevation coronary syndrome. In ischemic cerebrovascular disease, tirofiban was initially studied in patients with progressive stroke, and it was evaluated primarily with the European Stroke Scale (ESS) or National Institutes of Health Stroke Scale (NIHSS) in the patients whose symptoms worsened by 4 points within 72–96 h from onset of stroke (15, 16). Currently, tirofiban is recommended by many guidelines as an important short-term antithrombotic therapy for progressive stroke with arteriolar occlusion and perioperative patients undergoing endovascular intervention, but there are few clinical studies on its use in LHI. The purpose of this study was to investigate the efficacy and safety of tirofiban in the treatment of acute LHI.

In this prospective cohort study, the patients in the tirofiban group showed better neurological outcomes. The patients included in this study had similar baseline NIHSS scores. After treatment, a significant improvement in NIHSS score at discharge was seen in the tirofiban group compared with the baseline NIHSS score. There was a significant difference in NIHSS score reduction values at discharge between the tirofiban group and the control group The results of this study suggest that tirofiban can significantly improve the early neurological function in patients with LHI. This may be related to the unique way of administration of tirofiban. Tirofiban is administered intravenously and can rapidly increase the plasma concentration and greatly shorten the time to effect. The previous studies have shown that 90% platelet inhibition effect can be achieved within 30 min by intravenous administration (17). Meanwhile, as tirofiban is GPIIb/IIIa receptor antagonist that can block the final pathway of platelet aggregation, it is considered to be the most powerful platelet-specific inhibitor (18–21). In addition, LHI patients often demonstrate a level of consciousness preventing oral medication administration, thus requiring oral antithrombotic drugs to be crushed or melted and administered through a gastric tube. This may increase the risk of gastrointestinal bleeding caused by oral antithrombotic drugs, such as aspirin. In contrast, the intravenous administration of tirofiban has shown great advantages in avoiding gastrointestinal damage. In addition, the efficacy of clopidogrel can vary between patients. The previous studies have shown that patients with CYP2C19 allele deficiencies could not metabolize clopidogrel efficiently and the antiplatelet effect of clopidogrel was poor in that patients (22, 23). Compared with clopidogrel, tirofiban can directly play an antiplatelet effect without a need for liver metabolism.

**Figure 4:**
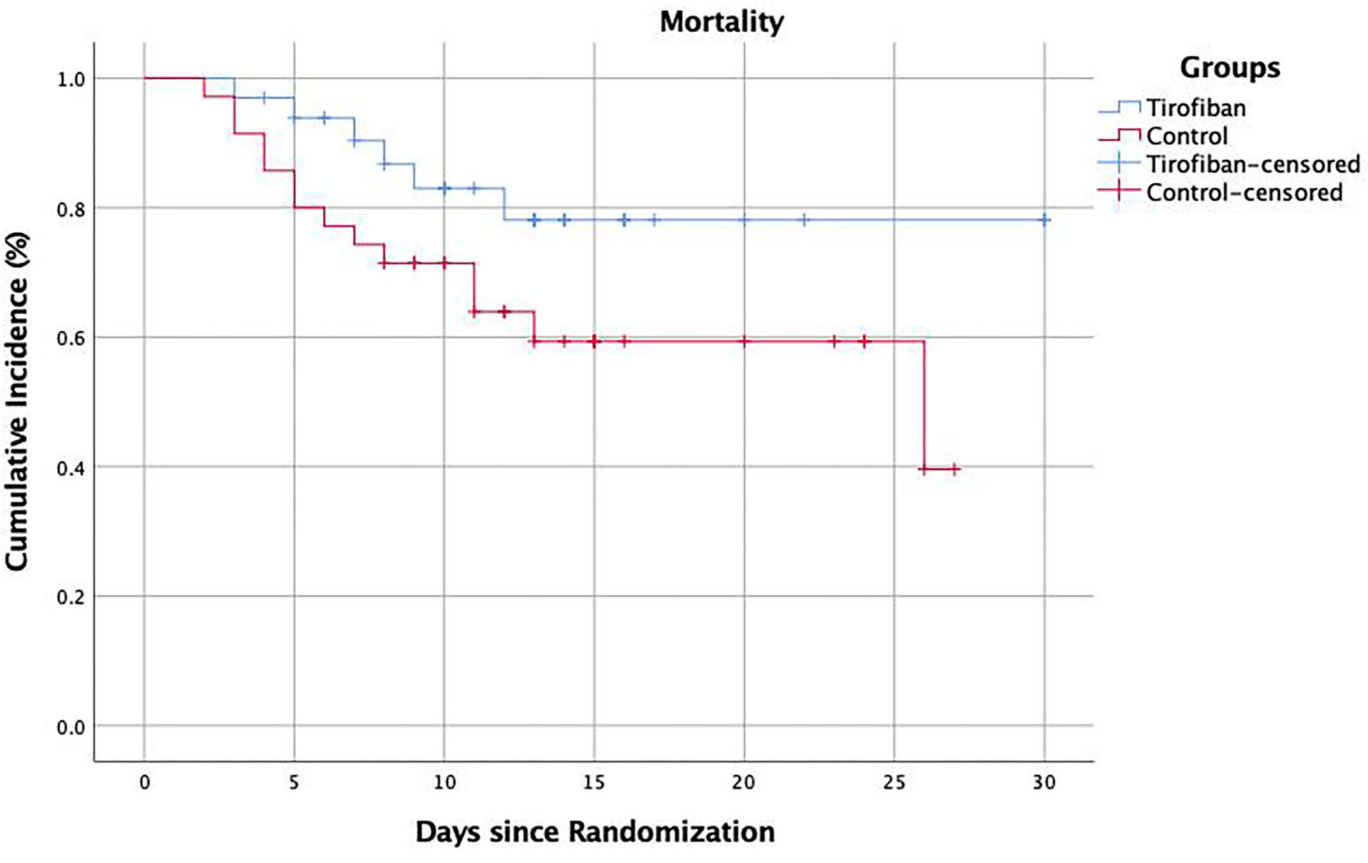
Cumulative mortality in hospital.

In addition, we found in the study that tirofiban significantly reduced the in-hospital mortality in patients. The Kaplan–Meier survival diagram showed that tirofiban significantly increased the in-hospital survival in patients with LHI compared with the control group (*p* = 0.046) (Figure 4). Of note, the subgroup analysis showed that the decrease in in-hospital mortality was more pronounced in the LHI patients older than 80 years. This result suggested that tirofiban was also safe and effective in elderly patients with LHI and may have greater clinical application value. The study also showed that tirofiban could significantly reduce the number of patients with mRS 5–6 scores at 90 days, which further verified the clinical effects of low-dose and long-term tirofiban in the treatment of LHI. However, the result was mainly caused by the difference in mRS 6, there was no significant difference in other mRS scores but only a tendency toward better functional outcomes which may be mainly due to the small sample size in this study.

In this study, we compared the safety of tirofiban in LHI patients by observing the rate of intracranial hemorrhage and other types of bleeding in the two groups. The results showed that compared with the control group, tirofiban did not increase the risk of symptomatic or asymptomatic intracranial hemorrhage. The risk of bleeding in the skin and mucosa system, digestive system respiratory system, and urinary system was also lower in the tirofiban group than that in the control group, which fully verified the safety of low-dose and long-term tirofiban in the treatment of acute LHI. Similar to this study, a number of studies have shown that tirofiban can effectively prevent vascular occlusion without increasing the risk of intracranial or non-intracranial hemorrhage in AIS patients 24 h after intravenous thrombolytic therapy with alteplase (6, 24). This result may be associated with the drug pharmacokinetics. The previous studies have shown that tirofiban's half-life is short (~1.6 h) and its inhibiting effect on platelet GP II B/III A receptor is reversible, so the antiplatelet effect can disappear 4 h after drug withdrawal in patients with a prolonged bleeding time (25–27). When there were signs suggesting bleeding events, the administration of tirofiban could be stopped immediately. In this study, the continuous administration of low-dose tirofiban was effectively reduced the risk of bleeding in LHI patients. On the whole, low-dose and long-term tirofiban may be safe in patients with LHI.

However, there were some shortcomings in this study. First, the sample size included in this study was small and may affect the reliability of the study. Second, the subjects enrolled in this study were only Han Chinese people, the efficacy and safety of low-dose and long-term tirofiban in LHI patients from other populations are still unclear. Third, the inconsistent baseline characteristics in the study may affect the study results to a certain extent. Meanwhile, this study only investigated the efficacy and safety of tirofiban at the dose of 3–4 μg/(kg·h) and the quantitative–effect relationship of tirofiban could not be evaluated.

In conclusion, low-dose and long-course tirofiban may significantly improve the early neurological function of patients with LHI and can reduce the in-hospital mortality, especially in patients >80 years old. It also may improve the quality of life and ability of daily living at 90 days. At the same time, tirofiban does not increase the risk of intracranial hemorrhage and another systemic bleeding. In view of some of the shortcomings of this study, a large number of high-quality clinical studies are needed to further clarify the clinical efficacy and safety of tirofiban in the treatment of acute LHI.

## Data availability statement

The raw data supporting the conclusions of this article will be made available by the authors, without undue reservation.

## Ethics statement

The studies involving human participants were reviewed and approved by the Ethics Committee of Putuo Hospital Affiliated to Shanghai University of Traditional Chinese Medicine. The patients/participants provided their written informed consent to participate in this study. Written informed consent was obtained from the individual(s) for the publication of any potentially identifiable images or data included in this article.

## Author contributions

YH was responsible for designing the experiment, collecting and statistics the data, and writing the manuscript. QX was responsible for collecting the data. ZS, YH, and ZC were responsible for the clinical treatment of patients. JC and GL were responsible for designing experiments, technical guidance, and editing manuscript. All authors contributed to the article and approved the submitted version.

## Funding

This work received financial support from the Construction Project of Specialized Clinical Diseases in the Health System of Putuo District, Shanghai (2019tszb02), Putuo District Health System Science and Technology Innovation Project (ptkwws201902), and Training Plan of 100 professionals in Shanghai Putuo District Central Hospital (2022-RCJC-05).

## Conflict of interest

The authors declare that the research was conducted in the absence of any commercial or financial relationships that could be construed as a potential conflict of interest.

## Publisher's note

All claims expressed in this article are solely those of the authors and do not necessarily represent those of their affiliated organizations, or those of the publisher, the editors and the reviewers. Any product that may be evaluated in this article, or claim that may be made by its manufacturer, is not guaranteed or endorsed by the publisher.
